# Effects of Return Air Inlets’ Location on the Control of Fine Particle Transportation in a Simulated Hospital Ward

**DOI:** 10.3390/ijerph191811185

**Published:** 2022-09-06

**Authors:** Jianlin Ren, Shasha Duan, Leihong Guo, Hongwan Li, Xiangfei Kong

**Affiliations:** 1 School of Energy and Environmental Engineering, Hebei University of Technology, Tianjin 300401, China; 2 Tianjin Jin’an Thermal Power Co., Ltd., Tianjin 300130, China; 3 Department of Biosystems & Agricultural Engineering, College of Engineering, Michigan State University, East Lansing, MI 48824, USA

**Keywords:** return air height, TOPSIS evaluation method, operating cost-effectiveness, exposure risk

## Abstract

The COVID-19 pandemic has made significant impacts on public health, including human exposure to airborne pathogens. In healthcare facilities, the locations of return air vents in ventilation systems may have important effects on lowering airborne SARS-CoV-2 transmission. This study conducted experiments to examine the influence of different return air vents’ heights (0.7 m, 1.2 m, and 1.6 m) on the particle removal effects in a simulated patient ward. Three different ventilation systems were examined: top celling air supply-side wall return (TAS), underfloor air supply-side wall return (UFAS) and side wall air supply-side wall return (SAS). CFD simulation was applied to further study the effects of return air inlets’ heights (0.3 m, 0.7 m, 1.2 m, 1.6 m, and 2.0 m) and air exchange rates. The technique for order of preference by similarity to ideal solution (TOPSIS) analysis was used to calculate the comprehensive scores of 60 scenarios using a multi-criterion method to obtain the optimal return air inlets’ heights. Results showed that for each additional 0.5 m distance in most working conditions, the inhalation fraction index of medical staff could be reduced by about 5–20%. However, under certain working conditions, even though the distances between the patients and medical personnel were different, the optimal heights of return air vents were constant. For TAS and UFAS, the optimal return air inlets’ height was 1.2 m, while for SAS, the best working condition was 1.6 m air supply and 0.7 m air return. At the optimum return air heights, the particle decay rate per hour of SAS was 75% higher than that of TAS, and the rate of particle decay per hour of SAS was 21% higher than that of UFAS. The location of return air inlets could further affect the operating cost-effectiveness of ventilation systems: the highest operating cost-effectiveness was 8 times higher than the lowest one.

## 1. Introduction

Since December 2019, the severe respiratory syndrome coronavirus 2 (SARS-CoV-2) has spread widely, causing the coronavirus disease (COVID-19) [1,2]. Concerns about potential exposures to SARS-CoV-2 for healthcare workers that treat COVID-19 patients have heightened [3,4,5,6]. The exposure risks to airborne pathogens for medical staff may greatly increase when they are in patient wards, compared to the risks when they are in the hallway, office, and nursing station, etc. [7]. There are numerous studies on the investigations of ventilation systems on reducing the airborne transmission risk of SARS-CoV-2, such as a systematic review of literature suggesting that ventilation systems may have significant impacts on viral aerosol transmissions [8,9,10].

Furthermore, reduced levels of airborne SARS-CoV-2 can be dependent on the characteristics of ventilation systems (e.g., location, type, flow rate, etc.) [11,12]. In recent years, based on experimental and simulation methods, many studies have analyzed the impacts of ventilation and filtration systems on indoor air quality. Tian et al. studied the performance of three types of ventilation systems on the pollutant removal in an office: Mixed ventilation, Displacement ventilation (DV) and Stratum ventilation (SV) [13]. SV was shown to obtain the optimal performance in removing pollutants emitted from several tested locations, and, thus, improving the inhaled air quality, compared to mixed ventilation and DV [13]. In another study, Lin et al. conducted a numerical simulation to analyze the particle dynamics in a classroom which was ventilated by DV or SV, and occupants were monitored in multiple locations in the classroom [14]. The results also suggested that the air flow in different ventilation types has meaningful impacts on the fate and transport of particles. The concentrations of particulate matter in the breathing zone of SV were significantly lower than those of DV [14]. In addition, compared with other ventilation types, SV can optimize the levels of thermal comfort and pollutant concentration in the breathing zone [15].

However, previous studies have mainly focused on the influence of the locations of the supply air vent. Return air vents are less well studied. It has been recently reported that the return air inlets may have important impacts on certain ventilation and filtration systems. Lin et al. found that the location of return air vents could affect the air diffusion in the tiered ventilation and the cooling load of the air handling unit [16,17]. Subsequently, Lin et al. studied the influence of several return air vent positions on thermal comfort indoors, and the results showed that the comfort level was optimal when the return air vents were located on the same side as the supply air vents [18].

Some studies have also pointed out that placing return air vents on the side wall may potentially save energy for the system, compared to the other locations [19]. In addition, the heights of the return air vents could have significant impacts on the energy consumption of the ventilation system, thermal comfort for the occupants, and indoor air quality in the confined spaces [20]. For instance, using numerical simulation, Fan et al. investigated the effects of the height of the return air vents in the Under Floor Air Supply (UFAS) system [21]. The heights of return air vents in the UFAS systems in large spaces were also examined [22,23]. Heidarinejad et al. found that the height of the return air vents of UFAS should be above 1.3 m to optimize energy consumption, thermal comfort and indoor air quality [20].

Although these aforementioned studies investigated the height of the return air vents [24], most of them primarily focused on the UFAS, and other air supply types, such as the Top Air Supply (TAS) or Side Air Supply (SAS), have been less reported on. As far as the authors know, very few studies have conducted research on the influence of the filtration effects and energy consumptions, and most have analyzed the influence of the height of return air vents on the indoor air quality. In fact, determining the optimal air return height is a multi-attribute decision-making problem. There are many evaluation indicators, and many studies have analyzed these through simple charts [20,21,22]. There is no well-established theoretical method to determine the optimal air return height. In order to solve the multi-dimensional evaluation index decision-making problem, some studies proposed to use the technique for order of preference by similarity to ideal solution (TOPSIS) method to select the optimal settings for heating, ventilation and air conditioning applications [25]. TOPSIS is a multi-criteria decision analysis method, which identifies the “positive ideal solution” and “negative ideal solution” based on the geometric distance between the positive ideal alternative and the tested alternative. The weights of each criterion are calculated in the TOPSIS method. To determine the attribute weights in the TOPSIS method, the entropy weight method is usually used, and the determination of the weight and decision result does not involve any subjective preference. In addition, some studies have used the TOPSIS method to evaluate the optimal blade angle of supply air vents under multi-index evaluation [26] and the optimal height of return air of the impingement jet system was determined by the TOPSIS method, which included multiple performance indicators in the analysis in [27]. In this study, there were 60 simulated scenarios tested to obtain data, and two criteria were analyzed to identify the optimal height of return air vents using various ventilation systems: indoor air quality (e.g., particulate matter attenuation rate, inhalation fraction index, pollutant removal efficiency) and energy consumption (e.g., air conditioning energy consumption).

The main objective of this study was to identify the optimal height of return air in different ventilation types, using experiments and multi-criterion analysis (i.e., the filtration effect and energy consumption). To achieve the goal, firstly, the impacts of return air heights on indoor air quality were examined in a laboratory to simulate a hospital ward using the following different factors: (1) air exchange rates; (2) supply air types; (3) and distances between the modeled medical staff and modeled patient. In addition, the energy consumption was calculated in each scenario, and the optimal working condition was identified by the TOPSIS method to establish a single ward environment that met the requirements of both air quality and energy saving.

## 2. Materials and Methods

### 2.1. Experimental Method

#### 2.1.1. Experimental Setup

**Figure 1:**
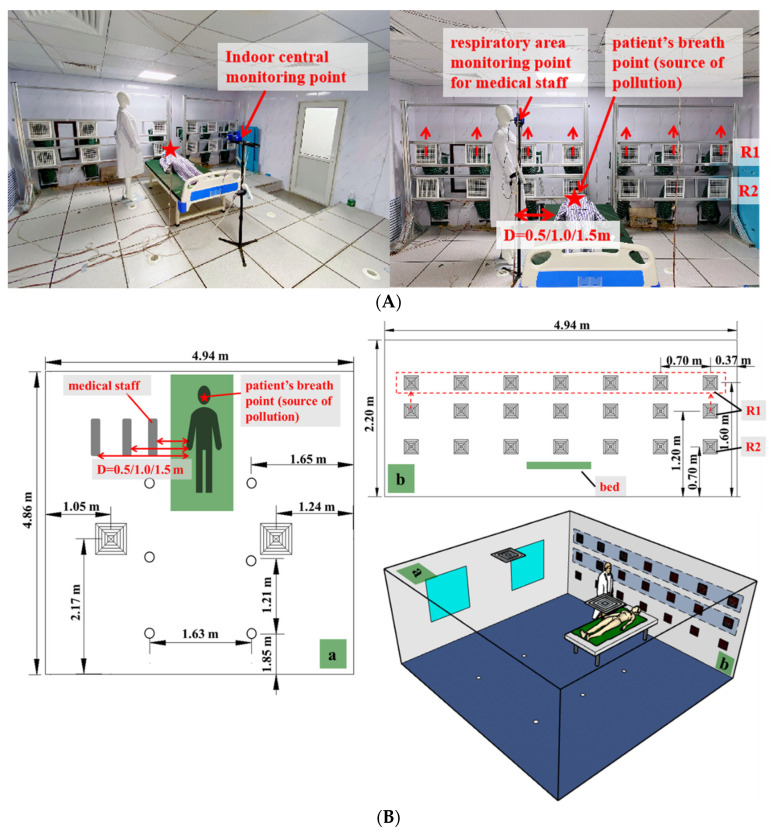
Schematic diagram of experimental chamber. (**A**) The layout of the central measurement point, medical staff measurement point, pollution source release point and return air inlets in the laboratory; (**B**) Dimensions of the experimental chamber.

In this study, the experimental single patient room shown in Figure 1A,B was assumed to be the intensive care unit (ICU), according to the requirements of National Standard Infectious Disease Hospital Building Design Code and Code for Design of General Hospital (G.B. Chinese. 2014a; G.B. Chinese. 2014b) [28,29]. The dimension of the experimental cabin was 4.94 m (length) × 4.86 m (width) × 2.20 m (height). The walls, floors, and ceilings of the experimental chamber were well insulated to obtain an appropriate thermal insulation performance. The temperature was maintained at 24 ± 1 °C and was continuously monitored by a T-type thermocouple (with an accuracy of ±0.1 °C in the range of −200 °C to 260 °C). A hospital bed with the size of 2.10 m (length) × 0.90 m (width) × 0.53 m (height) was placed near the wall, as shown in Figure 1. Particles were released from the mouth of the tested patient, who was lying on the back on the bed (height of 0.70 m). Particle concentrations were measured at two points: the breathing zone of the tested standing medical staff (height of 1.60 m) and the center of the room (height of 1.10 m) [15]. Two handheld airborne particle counters (TSI AEROTRAK MODEL 9306-V2, TSI, Inc., St. Paul., MN, USA) were used to monitor the particle concentrations. The shape and size (height of 1.80 m) of the two dummy models were identical, neither of them heated, to simplify the experiment. There were two square vents with a size of 0.5 m × 0.5 m on the top near the ceiling, six circular diffusers with a diameter of 0.9 m underfloor, and two rows on the side wall with a size of 0.2 m × 0.2 m square vents. The locations of the vents are shown in Figure 1B. The row near the ceiling was assigned as R1, the row near the underfloor was R2. The R1 vents could be moved. The distances from the center point of all air vents in R1 to the underfloor of the room could be adjusted from 1.2 m to 1.6 m (approximately to the breathing zone of medical staff). The distance from the center point of the square air vent in R2 to the underfloor of the room was 0.7 m (approximately to the breathing zone of the patient). To investigate the effects of the distance between medical staff and patient on the inhalation dose of particulate matter by medical staff, three distances were tested [30], which were 0.5 m, 1.0 m, and 1.5 m, respectively. It should be noted that in this study the medical staff were assumed to stand at the side of the bed. In fact, the locations of medical staff may also be different in addition to the distance. For example, medical staff usually stand at the foot of the patient’s bed during patient rounds. The effect of location of medical staff on their exposure risk will be discussed in further studies.

#### 2.1.2. Experimental Method

**Figure 2:**
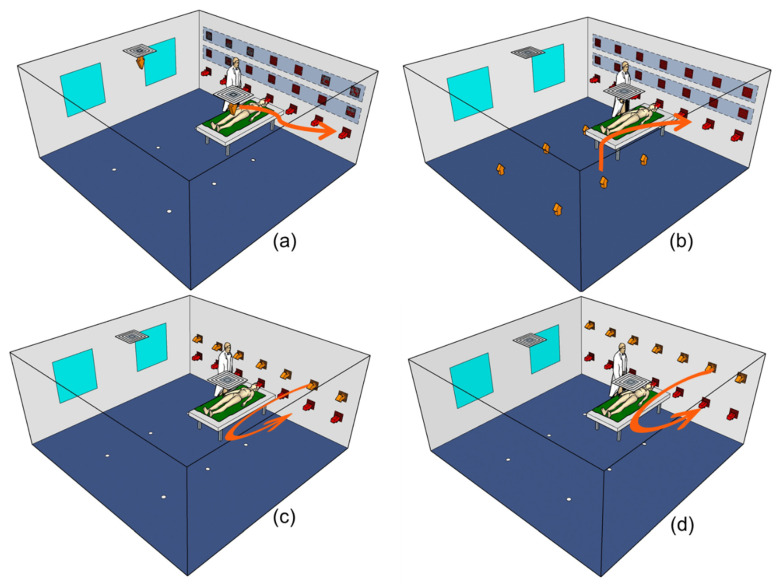
Schematic diagram of three air supply methods, in which the upper two return air outlets can be moved ((**a**) Top supply side return; (**b**) Underfloor air supply side return; (**c**) Side supply side return, 1.2 m air supply and 0.7 m air return; (**d**) Side supply side return, 1.6 m air supply and 0.7 m air return), the orange arrow represents supply air, and the red arrow represents return air.

According to the setup of the experimental chamber, three air supply forms were examined: TAS, UFAS, and SAS. On the basis of a pervious study [15], wherein Kong et al. found better performance of particle removal when the return air vents were on the side wall, compared to the removal levels using other air return modes, the return air vents of TAS, UFAS, and SAS systems were all located on the side wall in this study. As shown in Figure 2, there were three types of side air return heights for TAS and UFAS, which were 0.7 m, 1.2 m, and 1.6 m. The heights of the return air inlets of SAS were consistent at 0.7 m; the supply air outlets’ heights were set as 1.2 m and 1.6 m in two scenarios. The characteristics of the experimental parameters are listed in Table 1. The air exchange rate per hour (ACH) was tested at 6 and 12 in all scenarios [31]. According to the previous study [15], the draft sensation could be ignored under these air exchange rates for the three different ventilation systems.

Coil incense combustion mainly produces particulate matter, in addition to volatile organic compounds (VOC), carbon oxides (CO), nitrogen oxides (NO) and hydrocarbons (CH) [32]. The particles are spherical in shape. The density of the particles released when the incense burned was 900 kg/m^3^, and the mass concentration was 2 × 10^7^ μg/m^3^ [15]. The main aerodynamic diameters of the particles produced by the combustion of coil incense were between 0.3 and 10.0 μm [15] to simulate aerosol transmission. The combustion of the incense was maintained at the mouth of the patient to release a certain amount of dust to simulate indoor particulate matter. At the same time, the fan and particle counter both started to operate. During the experiment, the particle concentration between 0.3 and 10.0 μm in the mouth of the medical staff and the indoor center point were monitored and continuously recorded under different scenarios. Strict QA/QCs were conducted to ensure that the same amounts of particulate matter were produced [33].

### 2.2. Simulation Method

#### 2.2.1. Model Development

Considering the limitations of the experimental conditions, a Computational Fluid Dynamics (CFD) numerical simulation was carried out to further study the influence of different parameter settings on the control effect. Referring to a previous article [27], the heights of the return air vents were calculated at 0.3 m, 0.7 m, 1.2 m, 1.6 m and 2.0 m. In addition, the air exchange rates ranged from the original 6 ACH and 12 ACH to 6 ACH, 9 ACH, and 12 ACH [31]. A distance between the medical staff and the patient of 0.5 m was selected as representative for CFD simulation. Supplementary Table S1 summarizes all the simulated scenarios.

**Figure 3:**
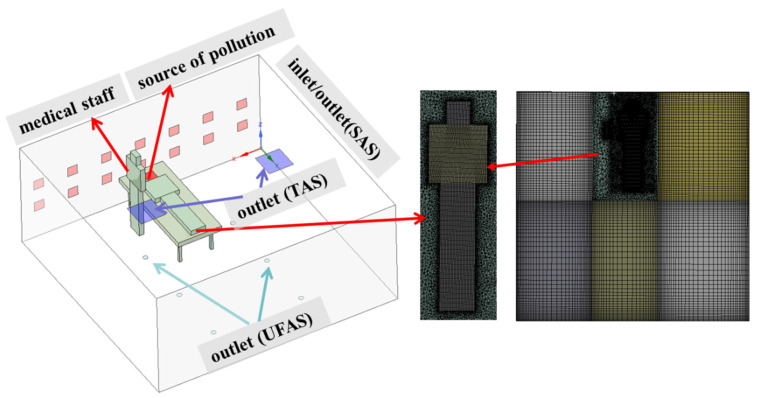
Schematic diagram of the CFD model and mesh.

As shown in Figure 3, an Ansys Design Modeler was used to establish a geometric model that simulated the air and pollutant distribution in a single ward. The size and location of the air vents in the room were identical to those of the experimental chamber. In the model shown in Figure 3, the two blue air inlets on the ceiling were TAS outlets; the six light blue circular air vents on the floor were UFAS outlets; the red air vents on the side wall were SAS supply air outlets and return air inlets. Particles were released from the red area of the patient’s mouth.

#### 2.2.2. Research on Grid Division and Grid Independence

The room was divided into multiple areas, and most of the computational domain was in structured grids. The modeled human body was surrounded by unstructured grids. To simulate the ventilation system around complex geometric shapes, unstructured grids may be used for processing, but more computational capacity and significantly longer calculation time were usually needed, compared to structured grids. Considering the accuracy of the simulation calculation of aerosols around the inlet and outlet and the wall boundary, the grid was densified at the wall boundary and the vents, and the grid was completed by mesh software (as shown in Figure 3).

**Figure 4:**
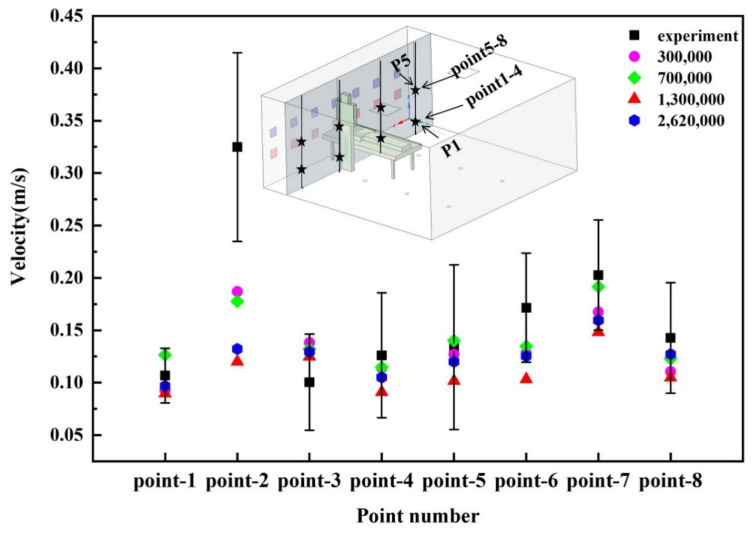
Grid independence study.

The grid independence of eight measuring points (the distance from the measuring point to the side wall near the patient’s head was 0.68 m, and the heights were 0.7 m and 1.2 m, respectively) was studied, and the number of grid nodes were 300,000, 700,000, 1.3 million, and 2.62 million, respectively. The grid quality was evaluated by selecting the skewness. The skewness value range was 0–1. The smaller the skewness value, the higher the grid quality. Maximum skewness values of the above four grids were all below 0.9, and the average skewness value was around 0.2. Generally, the quality of the four grids was acceptable. The ANSYS Fluent software was used for CFD simulation. The simulated velocity in the eight points was compared to the experimental velocity obtained in the previous study [15] under the SAS condition (1.2 m supply air and 0.7 m return air), and plotted in Figure 4. Except for point 2, the simulated air velocity in other points were within the allowable error range of the experiment. The 700,000 grids could not only ensure the simulation accuracy, but also saved calculation time and computer memory. Therefore, 700,000 grids were identified for the calculations.

#### 2.2.3. Model Verification

The detailed modeling methods were as follows: a three-dimensional ventilation system field, based on the RNG k-ε model; standard wall functions; SIMPLE algorithm to couple the pressure field and velocity field. In this analysis, the convergence criteria were set as 10^−5^ for the energy equation and 10^−4^ for other equations. In this study, the Lagrangian method [34] was used to simulate the behavior of particulate matter in the indoor space, and a DPM model was established. The patient’s mouth was set as the injection surface, and the particles were released transiently. Since the diameter of particles measured in the experiment was mostly distributed in the range of 0.3 μm–0.5 μm, the particle size was set as 0.4 μm in the simulation. The patient’s breathing rate was set as 0.8 m/s [35]. The number of particle flows was 2000. The random walk model was examined. The exit boundary was set to escape, and the remaining walls were trapped. The indoor temperature was at a constant temperature of 24 °C, and the inlet wind speed was calculated based on the air exchange rate.

**Figure 5:**
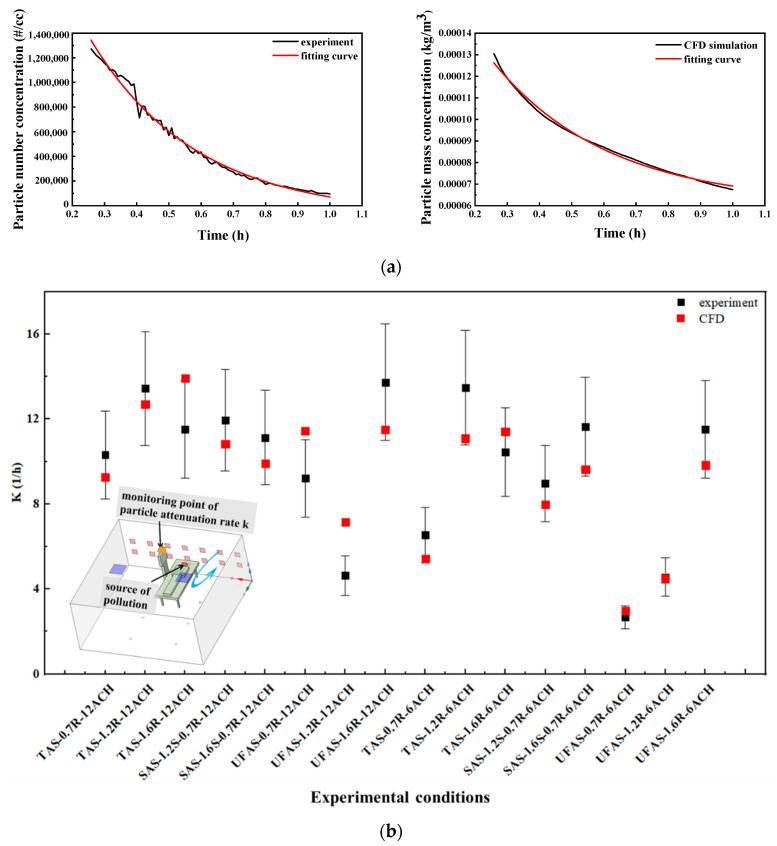
Model verification results. (**a**) Comparison of decay rate between experiment and simulation for one example case (SAS-1.2 m supply-0.7 m return-12 ACH); (**b**) Comparison of experimental and simulation results for 16 conditions.

The CFD models were verified using the experimental results in this study. To explain the model verification process, an example of the SAS system was described as below. The supply air vents and return air vents were at 1.2 m and 0.7 m from the floor, respectively. The room was ventilated at 12 ACH. The distance between the medical staff and the patient was 0.5 m. The concentrations of the particulate matter at the mouth of the medical staff were monitored and applied to calculate the particle concentration decay rate k. As shown in Figure 5a, the experimental value of the particle concentration decay rate k in the left picture was 11.94 and the simulated value in the right picture was 10.82; the error was within 20%. The validation of the model of this aforementioned particular scenario was acceptable. Similarly, the validation results of other experimental conditions are shown in Figure 5b. The distance between medical staff and patients in all experimental conditions was consistently 0.5 m. For most scenarios, the modeled decay rate was within 20% of the experimental error, suggesting that the model validation was acceptable.

#### 2.2.4. TOPSIS Evaluation Method

This study used the TOPSIS evaluation method which is based on the entropy weight method to comprehensively evaluate pros and cons of the simulated operating conditions. The TOPSIS model is essentially a ranking method, belonging to the multi-objective decision-making method. Its basic principle is to find out the optimal scheme and the worst scheme (expressed by positive ideal solution and negative ideal solution) in the limited scheme in the original matrix based on normalization, and then calculate the distance between the evaluation object and the optimal scheme and the worst scheme, respectively, so as to obtain the relative proximity (closeness) between the evaluation object and the optimal scheme, which is used as the basis for evaluating the pros and cons. The entropy weight method is used to calculate the actual data of samples (60 working conditions), which can eliminate the influence of human factors and obtain more objective results [36]. The combination of the entropy weight method and the TOPSIS model is widely used in all kinds of evaluation research [37,38].

**Figure 6:**
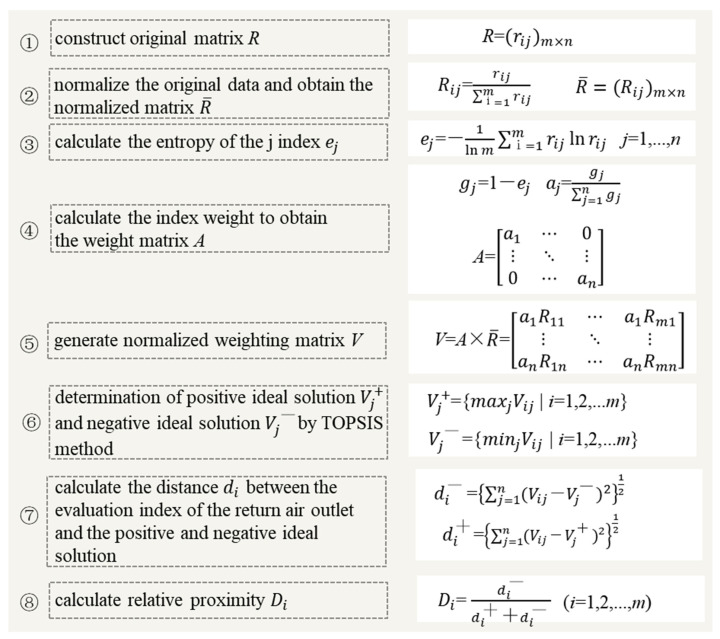
TOPSIS calculation process in this study.

There were four criteria included in the analysis: particle decay rate, inhalation score index, pollutant removal efficiency, and air conditioning energy consumption. These criteria are further discussed in the following section. Based on the results, the optimal heights of return air vents using various ventilation systems were identified. The value of the evaluation index closeness obtained from the final result was in the interval of [0, 1], and the closer its value was to 1, the better the evaluation object was. The specific steps of the TOPSIS method [27] are summarized and shown in Figure 6.

#### 2.2.5. Evaluation Index

To evaluate the performance of different ventilation systems more comprehensively, different evaluation indices were used in this study. The particle decay rate K was used to evaluate the removal of pollutants in the whole room. The inhalation fraction IF represented the distribution of particles near the mouth of medical staff, and was used to evaluate the distribution of local pollutants. The pollutant removal efficiency ε represented the ability of supply air to remove indoor pollutants and was used to determine the removal of indoor pollutants by supply air. Some other indices were also used in this study to compare the energy consumption of ventilation systems.

##### Particle Concentration Decay Rate k

The following equations were used to calculate the particle decay rate k [39]:

(1) $$c=c_{0}\times \exp\left(-\mathrm{kt}\right)$$

(2) $$k=k_{e}-k_{n}$$

where, c is the real-time concentration of fine particles, μg/m^3^; c_0_ is the initial concentration of particles, μg/m^3^; t is the decay time, h; k is the decay rate, h^−1^; k_e_ and k_n_ represents the total decay rate constant of the number of particles and the natural decay rate constant, h^−1^.

##### Inhalation Fraction Index IF

The inhalation fraction index is defined as the ratio of the mass concentration of a certain pollutant inhaled by an individual to the mass concentration released into the environment within a certain period of time [15]. The mouth of the medical staff was monitored for the mass concentration of particulate matter released into the environment and medical staff inhaled the mass concentration of particulate matter when turning on the fan.

(3) $$\mathrm{IF}=\frac{\int^{\;}Q_{b,\mathrm{inh}}c_{\mathrm{inh}}\mathrm{dt}}{\int^{\;}Q_{b,\mathrm{exh}}c_{\mathrm{exh}}\mathrm{dt}}$$

where, $Q_{b,\mathrm{inh}}$ and $Q_{b,\mathrm{exh}}$ are the respiratory rate of medical staff and patients (particle release point), respectively, L/min; ${\;c}_{\mathrm{inh}}$ is the average particle concentration inhaled by medical staff in the airway (here the average pollutant concentration in the mouth of medical staff when the fan is working), μg/m^3^; $c_{\mathrm{exh}}$ is the average mass concentration of particle released into the environment (here is the average pollutant concentration in the mouth of medical staff without a fan), μg/m^3^.

##### Particle Removal Efficiency ε

Particle removal efficiency [40] is a measure of the steady-state ventilation performance, which represents the ability of the system to remove pollutants. It can be calculated by the following equation:

(4) $$\varepsilon =\frac{c_{e}-c_{s}}{\;\bar{c}-c_{s}}$$

where, c_e_ is the particle concentration at the return air inlet, μg/m^3^; c_s_ is the particle concentration at the supply air outlet, μg/m^3^;  c ¯  is the average particle concentration in the room, μg/m^3^.

In this study, a HEPA filter was used in the air handling unit. Therefore, it can be assumed that the supply air was free of particles, and Equation (4) can be simplified as:

(5) $$\varepsilon =\frac{c_{e}}{\;\bar{c}}$$

In order to facilitate the evaluation of the particulate removal performance under different ventilation systems, the effectiveness of personal exposure ε_p_ [41] was calculated as follows:

(6) $$\varepsilon_{p}=\frac{c_{i,0}-c_{i}}{c_{i,0}-c_{\mathrm{pv}}}$$

where, c_(i,0)_ is the particle concentration in the air when a fan is off, μg/m^3^; c_i_ is the particle concentration inhaled by medical staff, μg/m^3^; c_pv_ is the particle concentration at the supply air outlet, μg/m^3^. In this study, it was assumed that the supply air was free of particles, and Equation (6) could be simplified as follows:

(7) $$\varepsilon_{p}=\frac{c_{i,0}-c_{i}}{c_{i,0}}$$

##### Energy Consumption of Ventilation System

The main energy consumption of ventilation systems consists of two variables: fan energy consumption and air handling energy consumption. The air handling energy consumption is the energy consumed by the air handling unit to process the air in order to eliminate the indoor residual heat and ensure the required indoor temperature and humidity. With a reference to the website [42], it was assumed that in summer, the outdoor temperature was 26 °C, relative humidity (RH) was 70%, and the enthalpy was 63.87 kJ/kg in Tianjin, China. In winter, the outdoor temperature was −2 °C, the RH was 50%, and the enthalpy value was 1.97 kJ/kg. According to the General Hospital Building Design Code [29], the indoor environmental conditions were suggested at 25 °C in summer were 55%RH, and enthalpy value 52.88 kJ/kg and at 22 °C in winter were, 55% RH, and enthalpy value 45.16 kJ/kg.

The fan energy consumption [43] was determined by:

(8) $$W_{\mathrm{fan}}=\frac{v△P}{\eta_{\mathrm{fan}}\eta_{\mathrm{motor}}}$$

where, W_fan_ is the energy consumption of the fan, W; v is the ventilation speed, m/s; ΔP is the average filtration pressure drop, Pa; η_fan_ is the fan efficiency, which can be assumed as 70% [43]; η_motor_ is the engine efficiency, which can be assumed as 65% [43]. A MERV 7 filter was used in this experiment, the formula ΔP = 735.1v^1.545^ was used to calculate the average pressure drop of the filter [43].

The air handling energy consumption was:

(9) $$W_{\mathrm{air}}=Q\rho_{\mathrm{air}}\left|i_{0}-i_{N}\right|$$

where, W_air_ is air handling energy consumption, W; Q is the flow rate of supply air, m^3^/h; *ρ*_air_ is the density of the air, 1.29 kg/m^3^; i_0_ and i_N_ are the air enthalpy values of the inlet and outlet, kJ/kg, the relevant data is listed above.

##### Operation Cost-Effectiveness of Ventilation Filter System

In this study, operating cost-effectiveness [44] was defined as the ratio of CADR to the energy consumption of the ventilation filter system, m^3^/kW·h; CADR = V × k, where V was the room volume, which was 52.8 m^3^ in this study, and k was the particle concentration decay rate, h^−1^.

## 3. Results and Discussion

### 3.1. Experimental Results

#### 3.1.1. Influence of the Distance between Medical Staff and Patients on Evaluation Indicators

**Figure 7:**
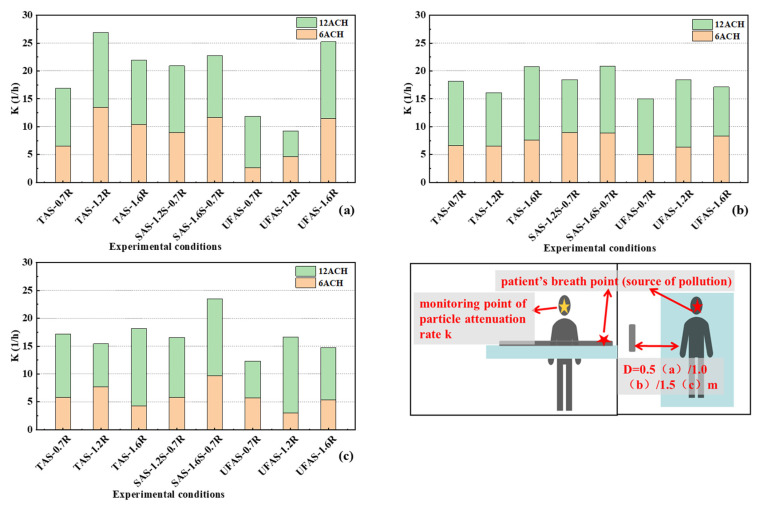
Particle concentration decay rate k at different distances between medical staff and patients. (**a**–**c**) represent the distance between the medical staff and the patient at 0.5 m, 1.0 m and 1.5 m, respectively.

Figure 7 shows the particle concentration decay rate k in the case of 6 ACH and 12 ACH when the distance between the medical staff and the patient was 0.5 m, 1.0 m, and 1.5 m, respectively. It can be seen from the figure that the decay rate of 12 ACH was larger than that of 6 ACH, indicating that the filtering effect of 12 ACH was better than that of 6 ACH. This finding agreed well with Lv et al. [45] and other studies [46] that showed that higher values of air exchange rate increased the removal rates of indoor pollutants.

When the distance between the medical staff and the patient was 0.5 m, TAS with return air vent height of 1.2 m was found to have better performance. At 6 ACH and 12 ACH, the particle decay rates K at this height were 13.44 h^−1^ and 13.48 h^−1^, respectively. UFAS with return air vent height of 1.6 m also performed well, at 6 ACH and 12 ACH, the particle decay rates K at this height were 11.50 h^−1^ and 13.73 h^−1^, respectively. SAS of 1.6 m air supply 0.7 m air return also performed well, at 6 ACH and 12 ACH, the particle decay rates K at this height were 11.64 h^−1^ and 11.12 h^−1^, respectively. When the distance between the medical staff and the patient was 1.0 m, the overall results were similar. TAS of 1.6 m return air had better performance, at 6 ACH and 12 ACH, the particle decay rates K at this height were 7.57 h^−1^ and 13.21 h^−1^, respectively. SAS of 1.6 m air supply 0.7 m air return also had better performance, at 6 ACH and 12 ACH, the particle decay rates K at this height were 8.85 h^−1^ and 12.01 h^−1^, respectively. When the distance between the medical staff and the patient was 1.5 m, SAS of 1.6 m air supply 0.7 m air return was better, at 6 ACH and 12 ACH, the particle decay rates K at this height were 9.72 h^−1^ and 13.76 h^−1^, respectively. To sum up, the distance between the medical staff and the patient had little effect on the decay rate under all scenarios. The performance of SAS of 1.6 m air supply 0.7 m air return was good at every distance. Some possible reasons may be: (1) the supply air height was identical with the height of the breathing zone of the medical staff in this scenario; (2) the filtration path under this ventilation system was reduced and, therefore, was beneficial for particle filtration.

**Figure 8:**
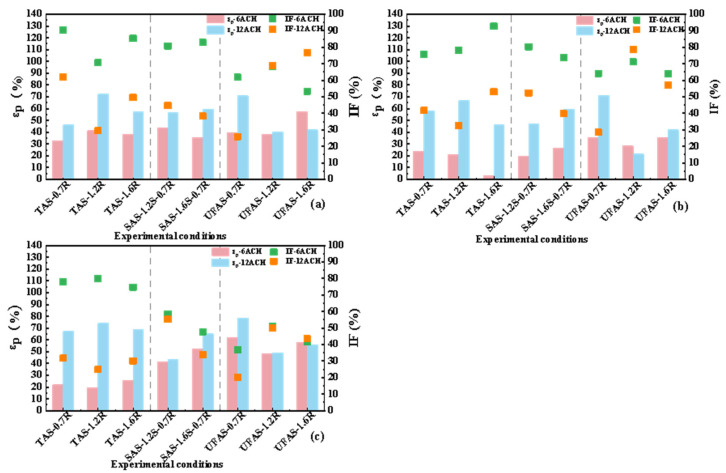
Inhalation fraction index IF and personal exposure effectiveness of healthcare workers at different distances between healthcare workers and patients. (**a**–**c**) represent the distances between the medical staff and the patient of 0.5 m, 1.0 m and 1.5 m, respectively.

Figure 8 shows the inhalation fraction index IF and personal exposure effectiveness ε_p_ of medical staff. The experimental conditions were: ventilation rates of 6 ACH and 12 ACH; distances between medical staff and patient of 0.5 m, 1.0 m and 1.5 m.

A lower value of the inhalation fraction index IF and a higher personal exposure effectiveness ε_p_ represented a lower exposure risk for healthcare workers. It can be seen from Figure 8 that the final results were almost the same under different distances. For TAS, the optimal height of return air vents was 1.2 m. For example, when the distance between the medical staff and the patient was 1.5 m, at 6 ACH and 12 ACH, the IF values of this working condition were 80.06% and 24.83%, respectively, and the ε_p_ values were 19.57% and 74.11%, respectively. For UFAS, the optimal height of return air vents was 0.7 m. For example, when the distance between the medical staff and the patient was 0.7 m, at 6 ACH and 12 ACH, the IF values of this working condition were 36.89% and 20.21%, respectively, and the ε_p_ values were 62.08% and 78.63%, respectively. The reasons may be the following. First, the dynamics of the particles released by the patient into the air may be impacted by SAS, which enhanced the accumulation of particles in the breathing zone of the medical staff. Second, TAS blows fresh air to the breathing zone of the medical staff, and quickly removes the particles exhaled by the patient through side return air method. Third, the side return air height of UFAS condition was 0.7 m, which was identical to the height of the patient’s breathing zone. Thus, the concentration of indoor particulate matter was lowered and the inhaled concentration of medical staff reduced.

When the distance between the medical staff and the patient was 0.5 m, the IF values were 53.36–90.48% and 25.92–76.68%, respectively and when the distance between the medical staff and the patient was 1.0 m, at 6 ACH and 12 ACH, the IF values were 64–92.8% and 28.57–78.65%, respectively. However, when the distance between the medical staff and the patient was 1.5 m, at 6 ACH and 12 ACH, the IF values were 36.89–80.06% and 20.21–55.34%, respectively. Figure 8a–c also show that the IF value of the 1.5 m distance was generally smaller than that of other distances. It shows that the distance between medical staff and patients influenced the IF and ε_p_ results. Longer distances may lower the infection risk of medical staff. In addition, for each additional 0.5 m distance in many working conditions, the inhalation fraction index of medical staff could be reduced by about 5–20% for most working conditions of 6 ACH. Zhou et al. [47] and Kang et al. [48] found a similar conclusion that the further distances were between people seated indoors, the better the pollutant removal effects and the lower the observed infection risks. Both the ventilation system and the location of indoor personnel impacted on the distribution of the pollutant field.

#### 3.1.2. Influence of Return Air Inlets’ Height on Evaluation Indicators

**Figure 9:**
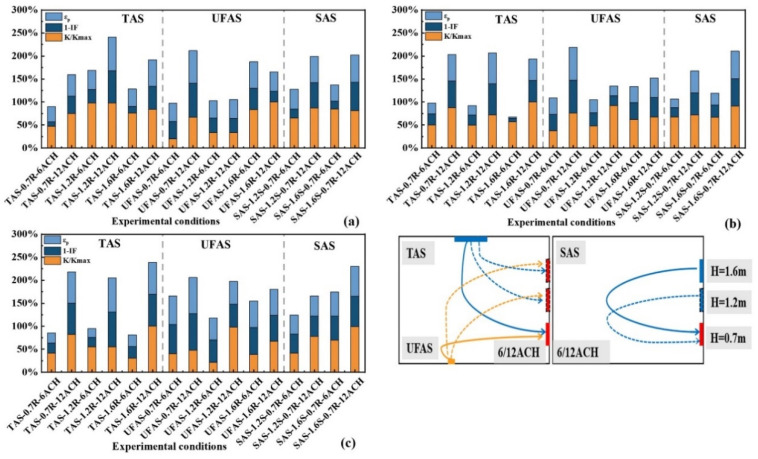
Evaluation indicators of different return air inlets’ heights at different distances between medical staff and patients. (**a**–**c**) represent the distances between the medical staff and the patient of 0.5 m, 1.0 m and 1.5 m, respectively.

The particle decay rate was tested as dimensionless to obtain K/K_max_. The larger K/K_max_ and ε_p_ and the smaller IF value indicated the better ventilation filtering effect. Higher values of 1-IF indicated better performance of ventilation systems. Figure 9a–c show that under identical conditions, a ventilation rate at 12 ACH could better improve the indoor air quality than at 6 ACH.

When the distance between the medical staff and the patient was 0.5 m (as shown in Figure 9a), the best return air height of TAS was 1.2 m, followed by 1.6 m. The best return air height of UFAS was 0.7 m, followed by 1.6 m. Due to the limited conditions of the experimental chamber, in SAS, the height of return air was only tested at 0.7 m, and the supply air heights were tested at both 1.2 m and 1.6 m. The results of 6 ACH and 12 ACH at the two different air supply heights were not much different. Whether it was 6 ACH or 12 ACH, the difference between ε_p_ and IF was smaller than 8% under the corresponding air change rates, and the difference between K value was smaller than 2.68 h^−1^. When the distance between the medical staff and the patient was 1.0 m (as shown in Figure 9b), the results of the three air return heights of TAS were similar. For UFAS, the return air height of 0.7 m had significant improvement. For SAS, 1.6 m air supply and 0.7 m air return condition was better. When the distance between the medical staff and the patient was 1.5 m (as shown in Figure 9c), the results of the three air return heights of TAS and UFAS were similar, while for SAS, 1.6 m air supply 0.7 m air return was better.

In summary, the comprehensive evaluation index results for different distances were similar. Based on the evaluation results of the three distances using TAS, the optimal return air height was at 1.2 m considering the exhaled particles of the patient, under the combined action of the thermal plume and the return ventilation system. For UFAS, the optimal return air height was 0.7 m, which was the same height as the patient’s breathing zone. For SAS, the supply air vents at 1.6 m and return air vents at 0.7 m showed the best performance. When the height of the supply air was identical to the height of the breathing zone of medical staff, and this was combined with a situation where the height of return air was identical to the height of the patient’s breathing zone, the performance was better than when the air was sent at a height of 1.2 m. The experimental results have implications for investigating the return air height to implement indoor air quality under different types of supply air.

Cheng et al. [49] suggested that the return air vents in a small space could be located in a lower area to save the energy of the cooling coil. Heidarinejad et al. [20] studied the effects of the heights of the side wall return air inlet and other parameters on the energy consumption, thermal comfort conditions and indoor air quality in UFAS, and found that reducing the height of return air inlet was more energy efficient. When the height of the return air inlet was 0.3 m, the energy consumption was the lowest. Due to the limited conditions of the experimental cabin, there were only three types of air return heights, and the minimum air opening of the experimental cabin was 0.7 m. Therefore, 60 scenarios were taken into further consideration by CFD simulation. The calculated evaluation indicators, as well as energy consumption, were used for TOPSIS evaluation.

### 3.2. Simulation Results

The evaluations were calculated for the energy consumption of ventilation and filtration systems [11,50,51,52] and the operating cost-effectiveness of the ventilation and filtration systems. The optimal return air heights for different types of supply air were subsequently identified, and, thus, the results can not only meet the air quality requirements, but also save energy. It can be seen from the experimental results that the comprehensive evaluation index results of different distances had little differences. Therefore, the distance between the medical staff and the patient of 0.5 m was selected as a representative for CFD simulation. Supplementary Table S1 shows all the simulated scenarios and the three air change rates included in each scenario. Based on the results of the simulation, the particle concentration decay rate k, the inhalation fraction index IF, and the pollutant removal efficiency ε were calculated for the analysis. All the calculated parameters are listed in the Supplementary Table S2.

Some studies [20] have reported the influence of the side wall return air inlet height and other parameters on energy consumption, thermal comfort conditions and indoor air quality in UFAS. It was found that reducing the height of the return air inlet can potentially be more energy efficient. When the height of the return air inlet was 0.3 m, the energy consumption was the lowest. Cheng et al. [49] also had a similar conclusion. Due to the limitation of the experimental chamber conditions, the minimum return air inlet height was 0.7 m. In CFD, the heights of return air vents were calculated at 0.3 m, 0.7 m, 1.2 m, 1.6 m and 2.0 m, to make up for the lack of experiments.

#### 3.2.1. Energy Consumption of Ventilation Systems

The air volume was calculated for various air change rates, and then these values were obtained for the energy consumption of fans and ventilation systems by means of Equations (8) and (9). Since the experiments were carried out in autumn and the indoor temperature and humidity should meet the requirements, calculating the fan energy consumption was only required for this analysis. The operating cost-effectiveness results and the TOPSIS method evaluation results in summer and winter are summarized in Figures S1–S4. It can be seen from Table 2 that as the air change rates enhanced, the energy consumption of the fan increased. At 6 ACH, the fan energy consumption was lowest at 102.3 W.

In hospitals, the ventilation systems are operated to filter particulate matter throughout the year. In spring and autumn, fan energy consumptions were solely considered for calculations, while in winter and summer, the sum of the energy consumption of the fan and the ventilation systems were included. As shown in Table 3, higher air change rates usually resulted in increased energy consumption. To maintain the air change rates in all seasons, the energy consumption in winter was much larger than that in summer. The lowest energy consumption was in spring and autumn, when the energy was 102.3 W and the ventilation system was operated at 6 ACH, while the highest energy consumption was 10.4 kW, when the room was ventilated at 12 ACH in winter.

**Figure 10:**
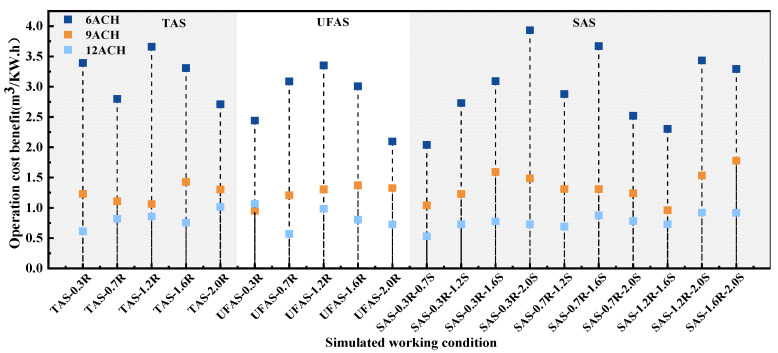
Operating cost-effectiveness of 60 simulated conditions.

The operational cost-effectiveness of ventilation filter systems was calculated according to Section of “Operation Cost-Effectiveness of Ventilation Filter System”. A sum of operating cost-effectiveness in 60 simulated operating conditions are shown in Figure 10. Compared with 9 ACH and 12 ACH, the operating conditions of 6 ACH showed better performance in energy saving. For TAS and UFAS, the cost-effectiveness of operation exceeded the others when the return air height was 1.2 m. Among the 60 operating conditions, the highest operating cost-effectiveness operating condition was SAS under the condition of 2.0 m air supply and 0.3 m air return with 6 ACH, the value being 3.93. The lowest operating cost-effective operating condition was SAS under the condition of 0.7 m air supply and 0.3 m air return with 12 ACH, the value being 0.53. The highest operating cost-effectiveness was about eight times higher than the lowest one.

#### 3.2.2. TOPSIS Evaluation Results

**Figure 11:**
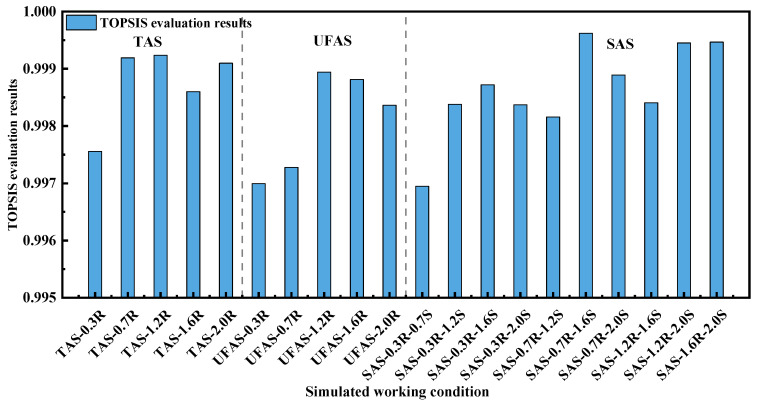
TOPSIS evaluation results.

By calculating the four evaluation indicators (i.e., particle decay rate k, inhalation fraction index IF, sewage efficiency ε, and operating cost-effectiveness), and using the TOPSIS evaluation method, the results of the 60 working conditions were obtained. The higher comprehensive scores suggested the better performance of the modeled scenarios. The weights of k, IF, ε, and the operating cost-effectiveness were 86.4%, 0.8%, 5.3% and 7.5%, respectively. (The specific calculation method is shown in Figure 6 where aj is the weight of the index j, and the larger the weight is, the more important the evaluation index is compared with other indices). Obviously, the weight of k was the highest amongst these indicators. Similar findings were also found in previous studies [53,54]. The evaluation results are shown in Supplementary Table S3. Since the evaluation results at 12 ACH were the highest amongst the ACHs, the results at 12 ACH are demonstrated in Figure 11. The return air heights at 0.7 m, 1.2 m, and 2.0 m had similar positive results in TAS, and the return air inlet’s height of 1.2 m was the best. For UFAS, 1.2 m was also the best height for return air inlet vents. For SAS, the best score was achieved by 1.6 m air supply and 0.7 m air return condition. The scores of the optimal scenarios under different types of supply air were significantly higher than that of other scenarios (especially for SAS), indicating that the optimal return air height of each supply air type obtained by the TOPSIS method had a reasonable reference value. Most of the scenarios with SAS and TAS had acceptable scores (higher than 0.998), while the scores with UFAS were relatively low. According to the results, the air quality at 12 ACH was better than that of 6 ACH, but the energy consumption was also higher. The results of air quality and energy consumption were negatively correlated, and, therefore, the determination of the best return air height should consider the comprehensive situations of indoor air quality and the energy consumption of the specific ventilation filter system.

Through this study, we clarified the relationship between the position of return air vents and the distance between the medical staff and the patient in the ward with experiments and CFD simulation. A multi-dimensional analysis (i.e., the filtration effect and energy consumption) was conducted to provide the best return air height for different air supply types in a single hospital ward. It can provide a reference for the design of ventilation and filtration systems, especially for infectious hospital wards.

### 3.3. Limitation

There are some limitations in this research that need further study. First, the human body model was not heated and the human body thermal plume could potentially affect the particle dynamics in the boundary layer between the human body and the bulk air. Second, both experiments and simulations were under static conditions, but in thereal world, the walking of the human body may also affect the experimental and simulation results. Third, according to the study by Kong et al. [15], the particle removal performance of ventilation systems with return air vents on the side wall had better performance than other return air modes. The results for ventilation systems with return air vents on other locations in the room need further investigations. Fourth, in the real hospital ward, the patients and medical staff may wear masks [55], which can also have effects on the IF of different ventilation systems. Last, this study focused on the control of particulate matter transportation in a simulated hospital ward with a ventilation method. In fact, an antivirus method may also be an effective method to control the concentrations of bacteria, viruses, etc. [9]. This also needs further study.

## 4. Conclusions

Experimental measurements and a modeling simulation analysis were conducted in this study. To obtain the optimal return air heights, three different types of supply air systems (i.e., TAS, UFAS, and SAS) were considered for simulation. The following findings were observed:

(1) Under different air supply types, even though the distances between the patients and medical personnel were different, the optimal heights of return air vents were constant. For TAS, the best performance was achieved by conditions with the return air inlet’s height of 1.2 m. For UFAS, the best performance was achieved by conditions with the return air inlet’s height of 0.7 m. For SAS, the best performance was achieved by the working condition of 1.6 m air return and 0.7 m air return.

(2) With the consideration of the effects of ventilation system on indoor air quality and energy consumption, the TOPSIS analysis results showed that the air change rate at 12 ACH was the best amongst all scenarios. For TAS and UFAS, the optimal return air inlet height was 1.2 m. For SAS, the best working condition was 1.6 m supply air and 0.7 m air return. The CFD simulation results also proved that after increasing the heights of the return air outlet, the performance of ventilation system was similar to the previous experimental results. 

(3) At the optical return air height, the particle decay rate per hour of SAS was 75% higher than that of TAS, and the rate of particle decay per hour of SAS was 21% higher than that of UFAS. Combined with the evaluation results of the three different air supply types by the TOPSIS method, it was suggested that SAS may be given priority, followed by TAS. This study can provide a reference for the future design of ventilation and filtration systems in hospital wards, so as to reduce the exposure risk of medical staff as much as possible.

## Figures and Tables

**Table 1:** Experimental air organization form and the height of the supply air outlets and return air inlets.

Air Supply Type	Return Air Inlets’ Height (m)
Underfloor air supply	0.7
Underfloor air supply	1.2
Underfloor air supply	1.6
Top air supply	0.7
Top air supply	1.2
Top air supply	1.6
Side air supply	1.2 supply and 0.7 return
Side air supply	1.6 supply and 0.7 return

**Table 2:** Energy consumption under different working conditions.

Air Exchange Rate (h^−1^)	Air Volume (m^3^/s)	Fan Energy Consumption (W)
6	0.088	102.3
9	0.132	287.1
12	0.176	569.9

**Table 3:** Energy consumption of ventilation filter system with different air changes in different seasons.

Air Exchange Rate (h^−1^)	Energy Consumption (kW)			
	Spring	Summer	Autumn	Winter
6	0.10	1.35	0.10	5.00
9	0.29	2.16	0.29	7.98
12	0.60	3.09	0.60	10.40

## Data Availability

The data that support the findings of this study are available from the author, upon reasonable request.

## Supplementary Materials

The following supporting information can be downloaded at: https://www.mdpi.com/article/10.3390/ijerph191811185/s1, Table S1: List of simulated working conditions; Table S2: Evaluation index values of 60 simulated working conditions; Table S3: TOPSIS evaluation index results for 60 simulated working conditions; Table S4: Draught rate(DR) calculation results; Figure S1: Operating cost-effectiveness of 60 simulated conditions in summer; Figure S2: Operating cost-effectiveness of 60 simulated conditions in winter; Figure S3: TOPSIS evaluation results in summer; Figure S4: TOPSIS evaluation results in winter.
