# Supplementary material for: Effects of Return Air Inlets’ Location on the Control of Fine Particle Transportation in a Simulated Hospital Ward
Source: Int J Environ Res Public Health. 2022 Sep 6;19(18):11185. doi: 10.3390/ijerph191811185 (PMC9517334; doi:10.3390/ijerph191811185)
Supplement: Supplementary file 1 [file ijerph-19-11185-s001.zip › ijerph-1823127-supplementary.pdf]

Supplementary S1:

Table S1. List of simulated working conditions.

Air supply type	Return air vent height (m)
Top air supply	0.3
Top air supply	0.7
Top air supply	1.2
Top air supply	1.6
Top air supply	2
Underfloor air supply	0.3
Underfloor air supply	0.7
Underfloor air supply	1.2
Underfloor air supply	1.6
Underfloor air supply	2.0
Side air supply	0.7 supply-0.3 return
Side air supply	1.2 supply-0.3 return
Side air supply	1.6 supply-0.3 return
Sde air supply	2.0 supply-0.3 return
Side air supply	1.2 supply-0.7 return
Side air supply	1.6 supply-0.7 return
Side air supply	2.0 supply-0.7 return
Side air supply	1.6 supply-1.2 return
Side air supply	2.0 supply-1.2 return
Side air supply	2.0 supply-1.6 return

Supplementary S2:

Table S2. Evaluation index values of 60 simulated working conditions

Air supply type	k	IF	ε
Side air supply-0.3 return-0.7 supply-6 ACH	3.952	0.912	0.558
Side air supply-0.3 return-0.7 supply-9 ACH	5.665	1.112	0.883
Side air supply-0.3 return-0.7 supply-12 ACH	6.013	1.720	0.898
Underfloor air supply-0.3 return-6 ACH	4.729	0.904	1.386
Underfloor air supply-0.3 return-9 ACH	5.162	1.264	0.963
Underfloor air supply-0.3 return-12 ACH	6.071	0.448	1.053
Top air supply-0.3 return-6 ACH	6.570	0.622	0.646
Top air supply-0.3 return-9 ACH	6.676	0.573	1.222
Top air supply-0.3 return-12 ACH	6.939	0.952	1.069
Side air supply-0.7 return-1.2 supply-6 ACH	5.580	0.808	3.774
Side air supply-0.7 return-1.2 supply-9 ACH	7.110	1.088	2.212
Side air supply-0.7 return-1.2 supply-12 ACH	7.820	0.749	1.679
Underfloor air supply-0.7 return-6 ACH	5.983	0.595	2.804
Underfloor air supply-0.7 return-9 ACH	6.551	0.697	1.588
Underfloor air supply-0.7 return-12 ACH	6.444	0.477	2.148
Top air supply-0.7 return-6 ACH	5.420	0.385	3.209
Top air supply-0.7 return-9 ACH	6.032	0.550	1.537
Top air supply-0.7 return-12 ACH	9.253	0.808	4.290
Side air supply-1.2 return-1.6 supply-6 ACH	4.461	0.436	0.771
Side air supply-1.2 return-1.6 supply-9 ACH	5.236	0.864	1.010
Side air supply-1.2 return-1.6 supply-12 ACH	8.252	0.528	1.316
Underfloor air supply-1.2 return-6 ACH	6.493	0.912	1.205
Underfloor air supply-1.2 return-9 ACH	7.092	1.712	1.121
Underfloor air supply-1.2 return-12 ACH	9.142	0.942	1.367
Top air supply-1.2 return-6 ACH	7.090	1.032	1.643
Top air supply-1.2 return-9 ACH	5.777	1.160	1.263

Top air supply-1.2 return-12 ACH	9.688	0.664	1.493
Side air supply-1.6 return-2.0 supply-6 ACH	6.380	1.576	1.057
Side air supply-1.6 return-2.0 supply-9 ACH	9.665	0.792	0.596
Side air supply-1.6 return-2.0 supply-12 ACH	10.370	1.120	1.668
Underfloor air supply-1.6 return-6 ACH	5.825	0.856	0.903
Underfloor air supply-1.6 return-9 ACH	7.458	1.320	0.407
Underfloor air supply-1.6 return-12 ACH	9.102	2.256	0.454
Top air supply-1.6 return-6 ACH	6.411	1.168	1.362
Top air supply-1.6 return-9 ACH	7.754	0.530	1.104
Top air supply-1.6 return-12 ACH	8.530	0.651	1.597
Underfloor air supply-2.0 return-6 ACH	4.057	0.896	0.414
Underfloor air supply-2.0 return-9 ACH	7.200	1.264	0.332
Underfloor air supply-2.0 return-12 ACH	8.191	0.500	1.268
Top air supply-2.0 return-6 ACH	5.251	0.598	0.663
Top air supply-2.0 return-9 ACH	7.084	1.368	0.681
Top air supply-2.0 return-12 ACH	9.493	1.800	1.139
Side air supply-0.3 return-1.2 supply-6 ACH	5.289	1.656	0.839
Side air supply-0.3 return-1.2 supply-9 ACH	6.687	1.464	1.361
Side air supply-0.3 return-1.2 supply-12 ACH	8.237	0.646	1.102
Side air supply-0.3 return-1.6 supply-6 ACH	5.989	1.144	1.611
Side air supply-0.3 return-1.6 supply-9 ACH	8.651	0.904	0.392
Side air supply-0.3 return-1.6 supply-12 ACH	8.774	0.880	1.226
Side air supply-0.3 return-2.0 supply-6 ACH	7.622	2.400	0.605
Side air supply-0.3 return-2.0 supply-9 ACH	8.067	0.968	2.138
Side air supply-0.3 return-2.0 supply-12 ACH	8.225	0.736	1.088
Side air supply-0.7 return-1.6 supply-6 ACH	7.110	1.600	2.778
Side air supply-0.7 return-1.6 supply-9 ACH	7.115	1.328	1.920
Side air supply-0.7 return-1.6 supply-12 ACH	9.893	1.392	4.312
Side air supply-0.7 return-2.0 supply-6 ACH	4.881	0.350	1.256

Side air supply-0.7 return-2.0 supply-9 ACH	6.748	0.344	2.285
Side air supply-0.7 return-2.0 supply-12 ACH	8.827	0.539	3.513
Side air supply-1.2 return-2.0 supply-6 ACH	6.655	0.767	1.695
Side air supply-1.2 return-2.0 supply-9 ACH	8.331	0.848	1.144
Side air supply-1.2 return-2.0 supply-12 ACH	10.452	0.399	1.568

Supplementary S3:

Table S3. TOPSIS evaluation index results for 60 simulated working conditions.

Air supply type	TOPSIS evaluation index results
Side air supply-0.3 return-0.7 supply-6 ACH	5.2E-05
Side air supply-0.3 return-0.7 supply-9 ACH	0.37352
Side air supply-0.3 return-0.7 supply-12 ACH	0.99694
Underfloor air supply-0.3 return-6 ACH	0.00056
Underfloor air supply-0.3 return-9 ACH	0.37352
Underfloor air supply-0.3 return-12 ACH	0.99699
Top air supply-0.3 return-6 ACH	0.00175
Top air supply-0.3 return-9 ACH	0.37353
Top air supply-0.3 return-12 ACH	0.99755
Side air supply-0.7 return-1.2 supply-6 ACH	0.00128
Side air supply-0.7 return-1.2 supply-9 ACH	0.37353
Side air supply-0.7 return-1.2 supply-12 ACH	0.99815
Underfloor air supply-0.7 return-6 ACH	0.00144
Underfloor air supply-0.7 return-9 ACH	0.37352
Underfloor air supply-0.7 return-12 ACH	0.99727
Top air supply-0.7 return-6 ACH	0.00113
Top air supply-0.7 return-9 ACH	0.37352
Top air supply-0.7 return-12 ACH	0.99919
Side air supply-1.2 return-1.6 supply-6 ACH	0.00035
Side air supply-1.2 return-1.6 supply-9 ACH	0.37352
Side air supply-1.2 return-1.6 supply-12 ACH	0.99840
Underfloor air supply-1.2 return-6 ACH	0.00171
Underfloor air supply-1.2 return-9 ACH	0.37353
Underfloor air supply-1.2 return-12 ACH	0.99894
Top air supply-1.2 return-6 ACH	0.00212
Top air supply-1.2 return-9 ACH	0.37352
Top air supply-1.2 return-12 ACH	0.99923

Side air supply-1.6 return-2.0 supply-6 ACH	0.00163
Side air supply-1.6 return-2.0 supply-9 ACH	0.37354
Side air supply-1.6 return-2.0 supply-12 ACH	0.99946
Underfloor air supply-1.6 return-6 ACH	0.00126
Underfloor air supply-1.6 return-9 ACH	0.37353
Underfloor air supply-1.6 return-12 ACH	0.99881
Top air supply-1.6 return-6 ACH	0.00166
Top air supply-1.6 return-9 ACH	0.37353
Top air supply-1.6 return-12 ACH	0.99859
Underfloor air supply-2.0 return-6 ACH	7.7E-05
Underfloor air supply-2.0 return-9 ACH	0.37353
Underfloor air supply-2.0 return-12 ACH	0.99836
Top air supply-2.0 return-6 ACH	0.00087
Top air supply-2.0 return-9 ACH	0.37353
Top air supply-2.0 return-12 ACH	0.99909
Side air supply-0.3 return-1.2 supply-6 ACH	0.00090
Side air supply-0.3 return-1.2 supply-9 ACH	0.37353
Side air supply-0.3 return-1.2 supply-12 ACH	0.99838
Side air supply-0.3 return-1.6 supply-6 ACH	0.00139
Side air supply-0.3 return-1.6 supply-9 ACH	0.37353
Side air supply-0.3 return-1.6 supply-12 ACH	0.99871
Side air supply-0.3 return-2.0 supply-6 ACH	0.00246
Side air supply-0.3 return-2.0 supply-9 ACH	0.37353
Side air supply-0.3 return-2.0 supply-12 ACH	0.99837
Side air supply-0.7 return-1.6 supply-6 ACH	0.00217
Side air supply-0.7 return-1.6 supply-9 ACH	0.37353
Side air supply-0.7 return-1.6 supply-12 ACH	0.99962
Side air supply-0.7 return-2.0 supply-6 ACH	0.00065
Side air supply-0.7 return-2.0 supply-9 ACH	0.37353

Side air supply-0.7 return-2.0 supply-12 ACH	0.99889
Side air supply-1.2 return-2.0 supply-6 ACH	0.00183
Side air supply-1.2 return-2.0 supply-9 ACH	0.37353
Side air supply-1.2 return-2.0 supply-12 ACH	0.99944

Supplementary S4: The operating cost-effectiveness results and the TOPSIS method evaluation results in summer and winter.

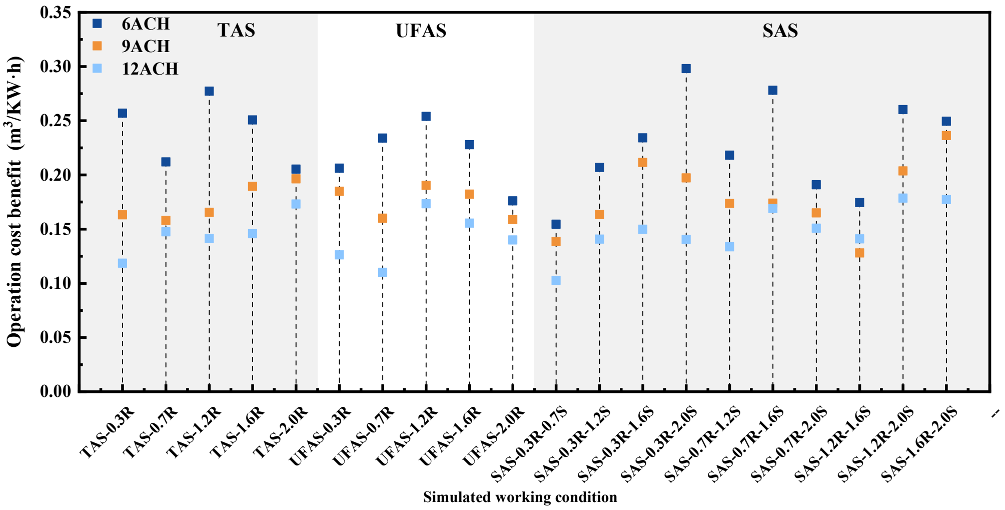

Figure S1. Operating cost-effectiveness of 60 simulated conditions in summer.

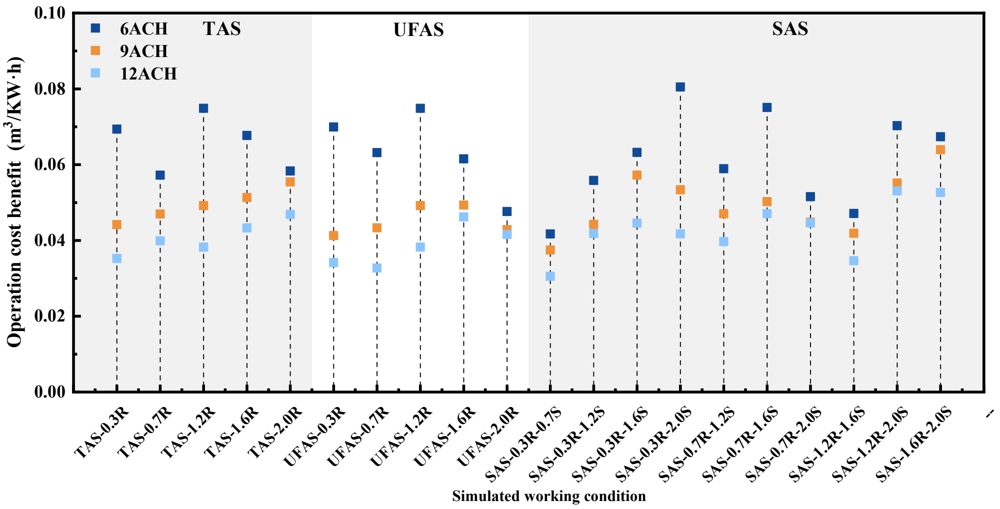

Figure S2. Operating cost-effectiveness of 60 simulated conditions in winter.

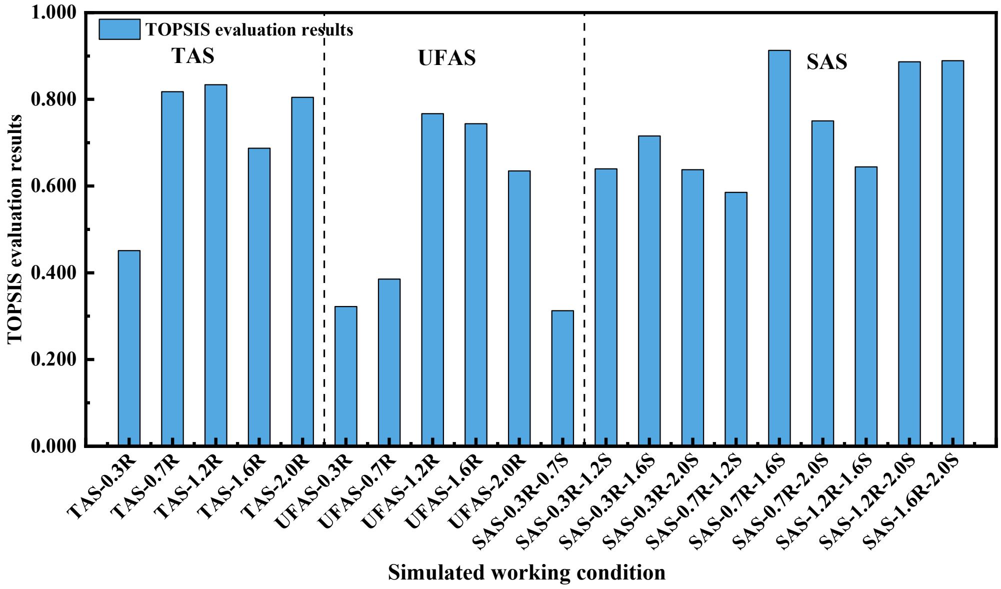

Figure S3. TOPSIS evaluation results in summer.

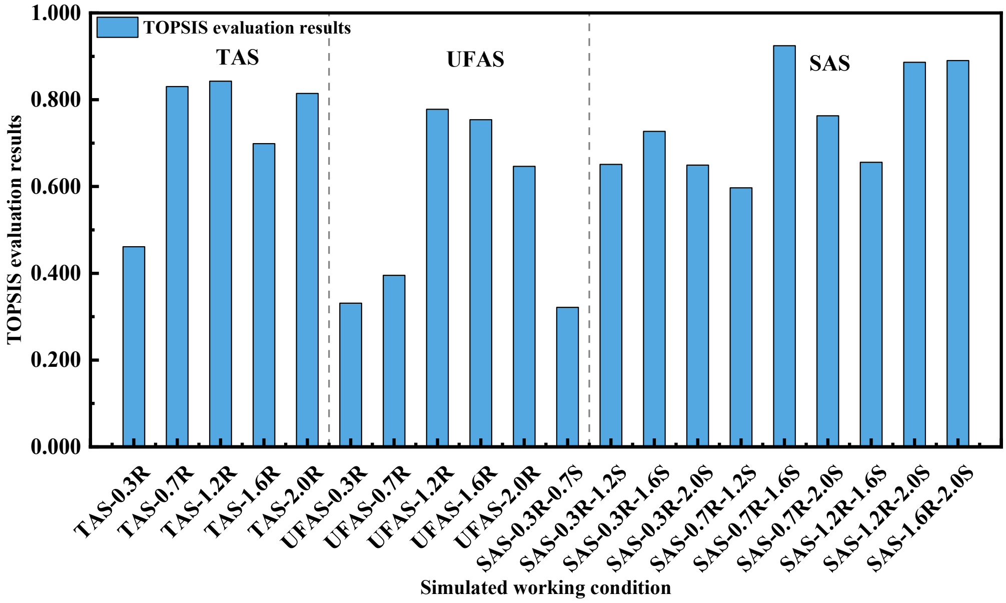

Figure S4. TOPSIS evaluation results in winter.
